# Cholesteryl Pullulan Encapsulated TNF-*α* Nanoparticles Are an Effective Mucosal Vaccine Adjuvant against Influenza Virus

**DOI:** 10.1155/2015/471468

**Published:** 2015-09-01

**Authors:** Daiki Nagatomo, Madoka Taniai, Harumi Ariyasu, Mutsuko Taniguchi, Miho Aga, Toshio Ariyasu, Tsunetaka Ohta, Shigeharu Fukuda

**Affiliations:** R&D Center, Hayashibara Co. Ltd., 675-1 Fujisaki, Naka-ku, Okayama 702-8006, Japan

## Abstract

We encapsulated tumor necrosis factor-*α* (TNF-*α*), a major proinflammatory cytokine, into cholesteryl pullulan (CHP) to prepare TNF/CHP nanoparticles. In this report, we describe the immune-enhancing capability of the nanoparticles to act as a vaccine adjuvant. TNF/CHP nanoparticles showed excellent storage stability and enhanced host immune responses to external immunogens. The nanoparticles were effective via the nasal route of administration for inducing systemic IgG_1_ as well as mucosal IgA. We applied the nanoparticles in a model experimental influenza virus infection to investigate their adjuvant ability. TNF/CHP nanoparticles combined with a conventional split vaccine protected mice via nasal administration against a lethal challenge of A/PR/8/34 (H1N1) influenza virus. Mechanistic studies showed that the nanoparticles enhanced antigen uptake by dendritic cells (DCs) and moderately induced the expression of inflammation-related genes in nasopharynx lymphoid tissue (NALT), leading to the activation of both B and T cells. Preliminary safety study revealed no severe toxicity to TNF/CHP nanoparticles. Slight-to-moderate influences in nasal mucosa were observed only in the repeated administration and they seemed to be reversible. Our data show that TNF/CHP nanoparticles effectively enhance both humoral and cellular immunity and could be a potential adjuvant for vaccines against infectious diseases, especially in the mucosa.

## 1. Introduction

In the last few decades, outbreaks of emerging infectious diseases such as severe acute respiratory syndrome (SARS) [1] and avian/swine influenza [2] impacted humanity's disease control systems to reconsider and have raised demand for the development of next-generation vaccines.

Vaccines are the most effective interventions against infectious diseases. Many vaccines, however, are effective only in preventing onset and aggravation of symptoms and less effective in preventing infection, in particular, respiratory infections such as influenza. Some reasons for this are that the major administration routes of conventional vaccines, including subcutaneous (*s.c.*) and intramuscular (*i.m.*) one, only induce neutralizing IgG antibody in blood and not mucosal IgA antibody, which is effective in preventing infection. In addition, the efficacy of IgG antibody against mutated viruses is very limited because it has highly restricted cross-protective capabilities. Conversely, IgA antibody on mucosa shows wide cross-protection and can block infection [3, 4]. When immunizations are delivered at the mucosa, IgA antibody is induced on mucosal surfaces throughout the body. As mucosal vaccination induces immunity in both the systemic and mucosal compartments [5, 6], enhanced antigen-specific mucosal immunity is a clear goal for next-generation vaccines. Mucosal and particularly nasal vaccines are promising because of their effectiveness in preventing infection via the respiratory tract. Nasal vaccines have the additional benefit of improved patient compliance and greater clinical convenience.

One significant drawback of mucosal vaccines is that they generally do not induce strong enough immune responses. The recent component-split vaccines, while avoiding many negative patient reactions, tend to be less immunogenic by themselves, even in the case of intravenous (*i.v.*) or* i.m.* administration. Generally, children and the elderly tend to respond less to vaccinations, which may lower the preventive power of the population [7]. Adjuvants must be administered simultaneously with the vaccine in order to enhance vaccine-specific immune responses. Among the adjuvants, Alum salts have been used the most, but they are neither suitable for all vaccines nor always capable of eliciting the desired immune responses. Other types of adjuvants have been under development, such as liposomes, emulsions, and their combinations [8, 9]. The development of safe, effective, and suitable adjuvants for various vaccines is expected to meet the new type vaccines.

Tumor necrosis factor-*α* (TNF-*α*) is a major proinflammatory cytokine primarily produced by T cells and macrophages [10]. Originally, it was found as a potent antitumor factor and is now known to play an integral role in host defense, including innate and adaptive immune activation, dendritic cell (DC) maturation, and subsequent T cell activation, as well as contributing to inflammatory responses [11, 12]. Interestingly, it was recently shown that TNF-*α* exerted adjuvant activities against pathogenic infections [13, 14]. Although the molecule has attracted the interest of many investigators and there were some attempts to develop TNF-*α* as a vaccine adjuvant, successful results have not been reported, probably because TNF-*α* causes unfavorable biological reactions when administered systemically and is rapidly degraded at mucosal surfaces when delivered mucosally. To overcome this, some investigators have attempted to generate protease-resistant mutant TNF-*α* molecules [15, 16].

In order to establish safer and more effective ways to administer bioactive substances, such as TNF-*α*, drug-delivery systems (DDS), nanoparticles made of biocompatible polymers, have attracted growing interest recently. One such material, cholesteryl pullulan (CHP), is the polysaccharide pullulan chemically modified with cholesteryl groups [17, 18]. It forms self-assembly nanoparticles in aqueous solution and entraps various molecules in its internal space through hydrophobic interactions. Furthermore, it protects the entrapped molecule from mechanical/chemical or enzymatic attacks outside the particle and acts as a superior carrier for delivery, achieving slow release of the encapsulated materials [19–21]. It has been shown that CHP nanoparticles are efficiently transferred to antigen-presenting cells such as macrophages and/or DC and that they elicit strong immune responses [22, 23]. CHP is under vigorous investigation for establishing novel vaccine therapies against several types of cancers [24–26].

In this report, we encapsulated human TNF-*α* into CHP resulting in TNF/CHP nanoparticles. Then, we examined the potential of the nanoparticles as a nasal vaccine adjuvant and protection against lethal influenza infection in a mouse model and conducted mechanistic analyses.

## 2. Materials and Methods

### 2.1. Human Tumor Necrosis Factor-*α* (TNF-*α*), Cholesteryl Pullulan (CHP), Influenza Virus, and Influenza Virus HA Vaccine (IVV)

Human TNF-*α* was prepared as previously described [27]. Briefly, TNF-*α* protein was produced by a human B cell lymphoblastoid cell line, BALL-1, stimulated with hemagglutinating virus of Japan (HVJ) and highly purified through a series of chromatography columns, including a specific monoclonal antibody column. The endotoxin level of the purified TNF-*α* was determined to be less than 300 pg/mg protein.

CHP is a partially (1–3% of glucose units) modified polysaccharide, pullulan, with cholesteryl residues (PUREBRIGHT CP-100T). It was purchased from NOF Co. (Tokyo, Japan).

Influenza virus A/Puerto Rico/8/34 (H1N1) strain was provided by Dr. Ayato Takada of the Research Center for Zoonosis Control, Hokkaido University, Japan. Influenza virus HA vaccine (IVV) “SEIKEN” was purchased from DENKA SEIKEN Co., Ltd. (Tokyo, Japan). It was a split and trivalent vaccine for seasonal influenza, consisting of the inactivated HA antigens A/Brisbane/59/2007 (H1N1), A/Uruguay/716/2007 (H3N2), and B/Brisbane/60/2008 (>30 *μ*g/mL each).

### 2.2. Preparation of TNF/CHP Nanoparticles

Two hundred fifty *μ*g/mL TNF-*α* and 12 mg/mL CHP were mixed, sterilized by filtration, and incubated at 37°C for 5 d. During the incubation, CHP encapsulated TNF-*α* molecules to form nanoparticles. Unencapsulated TNF-*α* was removed by size-exclusion chromatography with a PD-10 column (GE Healthcare, Fairfield, CT). The size of the particles was determined by dynamic light scattering (DLS) with a Zetasizer Nano ZS (Malvern Instruments Ltd., Malvern, UK). To estimate the amount of TNF-*α* encapsulated, nanoparticles were treated with 100 mg/mL methyl-*β*-cyclodextrin (Me-*β*-CD, Sigma-Aldrich Co., Saint Louis, MO) at 37°C for 2 h to disrupt the particle structure and release TNF-*α*, as described [21]. The amount was determined by an enzyme-linked immunosorbent assay (ELISA) system described below.

### 2.3. TNF-*α* ELISA

The amount of human TNF-*α* was determined by an ELISA system established in-house in Hayashibara Co., Ltd. Briefly, an anti-human TNF-*α*-specific mouse antibody (MAb-TNF-*α*-5) was immobilized in 96-well plates (NUNC-IMMUNO PLATE U96, Nunc, Rochester, NY). After incubating with the samples, horse radish peroxidase- (HRPO-) labeled anti-human TNF-*α*-specific mouse antibody (MAb-TNF-*α*-1-HRPO) was added to the wells. Then,* o*-phenylenediamine, a substrate for HRPO, was added to detect the immune complex and the absorbance at 490 nm was measured. Purified human TNF-*α* protein was used as the standard. This ELISA system recognized the active trimeric TNF-*α* molecule only and the detection limit was 0.2 ng/mL.

### 2.4. Animals

BALB/c mice (8-week-old females) were obtained from Charles River Laboratories Japan Inc. (Yokohama, Japan) and used after a week of quarantine and acclimation. This study was approved by the Laboratory Animal Care Committee of Hayashibara Co., Ltd., and all animal experiments and procedures were in accordance with the Guidelines for the Care and Use of Laboratory Animals at Hayashibara Co., Ltd.

### 2.5. Immunization of Animals

Mice were anesthetized with sevoflurane and the antigen and/or adjuvant preparations (15 *μ*L a nasal cavity, 30 *μ*L/mouse) were administered nasally at the indicated times.

### 2.6. Preparation of Blood, Nasal Wash, Nasopharynx-Associated Lymphoid Tissue (NALT), and Nasal Passage Cells

Blood and nasal wash were obtained from mice. Briefly, the animals were anesthetized with ether and blood was taken from the abdominal aorta with heparinized syringes. The jaw was incised bilaterally and the nasal cavity, in vicinity to root of the tongue, was exposed. The nasal cavity was washed with saline and the wash was recovered. The blood and the nasal wash were centrifuged at 5,000 rpm for 8 min and stored at −80°C until examination. Then, after thorough washing of the nasal cavity with saline, the upper palates were removed and the NALTs were isolated under the microscope. NALT cells were suspended with 100 *μ*m strainer (BD, Franklin Lakes, NJ) and recovered by centrifugation. The nasal passage cells were prepared as reported [28]. Briefly, the nasal passage cells were obtained by chipping off from the wall of the nasal cavity, minced, and dispersed with collagenase D (Roche, Basel, Switzerland). The cells were suspended with strainers and separated by biphasic centrifugation with 40% and 75% Percoll (GE Healthcare) at 1,000 rpm for 25 min. The cells in the boundary phase were collected and washed with RPMI 1640 medium.

### 2.7. IVV Antigen-Specific IgG_1_ and IgA Responses

Antibody responses in the plasma and the nasal wash of the immunized animals were determined using ELISA. IVV was immobilized in microplates and the respective antibody in the plasma or the nasal wash was examined. As the indicators, HRPO-labeled rat anti-mouse IgG_1_ (Life Technologies Co., Carlsbad, CA) for IgG_1_ and HRPO-labeled goat anti-mouse IgA (Southern Biotechnology Associates Inc., Birmingham, AL) for IgA were used with* o*-phenylenediamine. The absorbance was measured at 490 nm.

### 2.8. Hemagglutinin-Specific Antibody Responses

Specificity of antibody response was examined by detection of neutralizing activity in plasma against HAs of influenza type A H1N1, type A H3N2, and type B. Plasma from mice immunized with IVV and TNF/CHP nanoparticles were subjected to hemagglutination inhibition (HI) assay performed by FALCO biosystems Ltd. (Kyoto, Japan). The results were expressed as geometric mean titer (GMT).

### 2.9. Flow Cytometry Analyses of NALT Cells

Sixty-eight hours after the 3rd nasal immunization, NALT cells were prepared from mice as described. After being blocked with FcR blocker (Milteny Biotec, Bergisch Gladbach, Germany), the cells were stained with fluorescein isothiocyanate- (FITC-) labeled anti-mouse CD11c antibody and FITC-labeled anti-mouse CD80 antibody, or FITC-labeled anti-mouse CD86 antibody (Milteny Biotec), and subjected to flow cytometry (FCM) analysis using a flow cytometer (GALLIOS, Beckman Coulter, Brea, CA).

### 2.10. Antigen Uptake by NALT Cells and Nasal Passage Cells

Ovalbumin (OVA, Sigma-Aldrich Co.) was labeled with Alexa 647 using the Alexa Fluor 647 labeling kit (Life Technologies Co., Carlsbad, CA) according to the manufacturer's instructions. The resulting Alexa-labeled OVA was used as an indicator antigen for nasal administration to mice with or without the TNF/CHP nanoparticles as an adjuvant. Six h after administration of 10 *μ*g labeled OVA, the NALT cells and the nasal passage cells were prepared from the mice as described above, blocked with FcR blocker (Milteny Biotec, Bergisch Gladbach, Germany), stained with phycoerythrin- (PE-) labeled anti-mouse CD11c antibody (eBioscience, San Diego, CA), and subjected to FCM analysis. The results were examined with a flow cytometry analysis software, Gatelogic (Inivai Technologies Pty Ltd., Mentone, Victoria, Australia). The antigen uptake was estimated by the florescence intensity of Alexa 647 against intensity of CD11c^+^ cells, activated dendritic cells (DCs).

### 2.11. Quantitative PCR Analysis

Total RNA was prepared from NALT cells pooled from 8 mice, approximately 4 × 10^6^ cells, using the RNeasy Mini Kit (Qiagen, Hilden, Germany) according to the manufacturer's instructions. After confirming the purity and quality of the RNA, cDNA was synthesized using the RT^2^ First Strand Kit (SA Biosciences Co., Frederick, MD). The expression of various cytokines and factors related to immune responses was examined with the mouse innate and adaptive immune responses PCR array (SA Biosciences Co.) with a Roche LyteCycler 480 (Roche) according to the instructions.

To further analyze the expression of factors related to innate and adaptive immune responses, primer sets for interleukin-1*α* (IL-1*α*), IL-1*β*, IL-2, IL-4, IL-5, IL-6, CXC ligand 2 (CXCL2), IL-10, IL-12*β*, IL-17*α*, IL-18, interferon-*β* (IFN-*β*), IFN-*γ*, TNF-*α*, fibronectin-1, CD14, toll-like receptor 3 (TLR3), proteoglycan-2, lipopolysaccharide (LPS) binding protein, and lactotransferrin (Table 1) were prepared (Sigma-Aldrich Co.). Expression levels were normalized with the level of GAPDH and quantified by quantitative PCR with a Roche LyteCycler 480 (Roche).

### 2.12. Cell Proliferation and Cytokine Production Analyses

The NALT cells pooled from 8 mice were cultured for 3 d in microplates with feeder splenocytes treated with 50 *μ*g/mL mitomycin for 30 min, in the presence of the IVV antigen. The cell proliferation was examined with alamarBlue (Life Technologies Co.) and represented the difference (Δ) of fluorescence intensity between cases with and without IVV. The culture supernatant was recovered at 16 h and the cytokine-producing cells were examined using the ELISpot PLUS mouse IL-4 and the ELISpot Plus mouse IFN-*γ* (Mabtech AB, Nacka Strand, Sweden) kits. The results represented the difference of the number (Δ) of cytokine-positive cells/10^6^ cells between cases with and without IVV stimulation.

### 2.13. Adjuvant Effect of TNF/CHP Nanoparticles with IVV against Lethal Influenza Virus Challenge in Mice

Mice were anesthetized with sevoflurane and were administered nasally the preparation (15 *μ*L per nasal cavity, 30 *μ*L/mouse) once a week for 3 weeks (*n* = 10). Samples were prepared by mixing 1 : 1 IVV and the nanoparticle preparation (5 *μ*g/mouse) or Cholera Toxin B-subunit (CTB) (0.8 *μ*g/mouse, Sigma-Aldrich Co.) before use. Seven d after the final immunization, the mice were challenged with influenza virus (Puerto Rico/8/34, 160 pfu/50 *μ*L) nasally. The dosage of virus corresponded to 10 LD_50_ for the mice. The virus-challenged mice were monitored daily for body weight and signs of morbidity. Mice that were moribund or that had lost more than 20% of their body weight were considered to have reached an experimental endpoint and were humanely euthanized by anesthetization with pentobarbital.

### 2.14. Preliminary Safety Study

In reference to the OECD guideline for the testing of chemicals [29], preliminary safety study was performed. Mice (4 heads each sex, each group) were administered nasally the TNF/CHP nanoparticle preparation combined with the IVV once a week either once or four times for acute or repeated toxicity, respectively. In addition, some animals went through 4 weeks of repeated administrations followed by 2 weeks of cessation and were examined. The general symptoms were examined once a day and body weights and food consumption were recorded weekly. Body temperatures were recorded at 0 d before and 2 h after the administrations. The other items (ophthalmic examination, hematology, blood biochemistry, urinalysis, blood antibody titer, autopsy findings, and histopathological analyses of major organs) were inspected at 3 d for acute toxicity, and 0, 24, and 37 d for repeated toxicity. Blood biochemistry analyses were performed by Oriental Yeast Co. Ltd. (Tokyo, Japan). Furthermore, nasal mucosal tissue, the administration site, was examined histopathologically.

### 2.15. Data Analysis and Statistical Procedures

All values are expressed as mean values ± SD or SEM. The Student *t*-test was used for comparison between 2 groups. One-way ANOVA with Tukey test was used to determine the significance of differences for multiple comparisons. Differences with a probability value of *P* < 0.05 were considered to be significant.

## 3. Results

### 3.1. Preparation of TNF/CHP Nanoparticles

**Figure 1:**
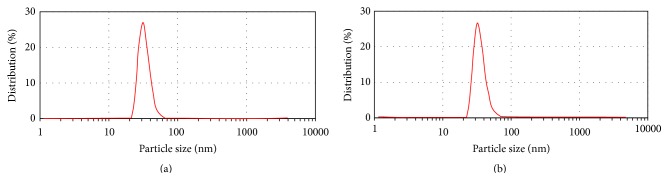
Size distribution of TNF/CHP nanoparticles. Particle size was determined by dynamic light scattering (DLS) with a Zetasizer Nano ZS (Malvern Instruments Ltd.). (a) TNF/CHP nanoparticles, (b) blank CHP particles.

CHP encapsulated active trimer TNF-*α* to form stable nanoparticles. Based on preliminary experiments, the optimal conditions for preparing the nanoparticles were incubating 250 *μ*g/mL TNF-*α* with 12.1 mg/mL CHP at 37°C for 5 d. The encapsulating process was temperature- and time-dependent and it hardly occurred in the 4°C conditions. Under these conditions, typically more than 90% of the TNF-*α* was encapsulated into CHP complexes, and the resulting nanoparticles were relatively uniform. The peak and average sizes of the particles were 27.2 nm and 42.4 nm based on the DLS results, not different from those of the blank CHP particle itself, 27.6 nm and 42.8 nm, respectively (Figure 1). Stoichiometric analyses showed that a TNF/CHP nanoparticle consisted of a TNF-*α* active trimer (ca. 50 kDa) in a CHP tetrameric complex (ca. 400 kDa).

### 3.2. Storage Stability of TNF/CHP Nanoparticles

Stability of the nanoparticles in Dulbecco's phosphate buffer (D-PBS) was evaluated at 25°C. To estimate the amount of encapsulated TNF-*α*, Me-*β*-CD was used to disrupt the CHP complex to release the TNF-*α* inside the particles. The results showed that the nanoparticle retained its integrity and kept TNF-*α* molecules active inside the complex in aqueous solution at room temperature for at least 21 d (Table 2). The integrity of the particles was also maintained more than 80% even after five cycles of freezing and thawing (data not shown). These results showed that the TNF/CHP nanoparticles were remarkably stable. However, upon contact with high concentrations of dissolved proteins, such as albumin in serum, the nanoparticles are prone to release the encapsulated TNF-*α* rapidly, probably being replaced with the proteins outside as reported [30] (data not shown).

### 3.3. Immune Responses Induced by TNF/CHP Nanoparticles Administered Nasally

**Figure 2:**
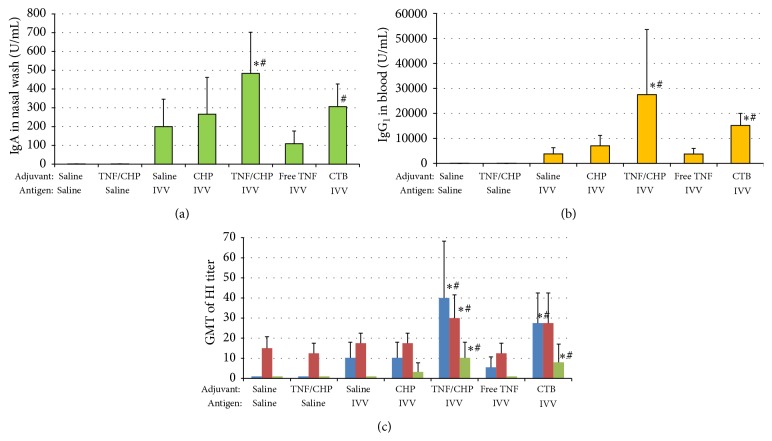
Adjuvant effects of TNF/CHP nanoparticles administered nasally. Mice were administered IVV (0.3 *μ*g/mouse) and TNF/CHP nanoparticles (5 *μ*g/mouse as TNF-*α*) or CTB (0.8 *μ*g/mouse) nasally once a week 4 times. The nasal wash and blood plasma were prepared from the mice and the levels of IVV-specific IgA and IgG_1_ were determined by ELISA. (a) IgA levels in nasal wash, (b) IgG_1_ levels in blood plasma, and (c) HI titer in blood plasma against different HA types of influenza virus. Blue column, type A/H1N1; red column, type A/H3N2; and green column, type B. (mean ± SEM, *n* = 8). ^*∗*^ *P* < 0.05 versus saline/IVV; ^#^ *P* < 0.05 versus free TNF/IVV.

Although TNF-*α* is known to have immune-enhancing activity [13], severe and unfavorable effects have hampered its practical use. We thought that delayed release of the TNF/CHP nanoparticle might promote the beneficial effects of TNF-*α*, such as a vaccine adjuvant, while avoiding harmful events. We examined the adjuvant activity of the TNF/CHP nanoparticles, for example, enhanced induction of antigen-specific antibodies in mice, particularly in the case of nasal administration. The results show that the nasally administered TNF/CHP nanoparticles combined with IVV induced significant levels of IgA in the nasal wash, as well as IgG_1_ in blood plasma (Figures 2(a) and 2(b)). These results are comparable to those of the positive control CTB, a model adjuvant [31]. However, IVV with CHP alone (without TNF-*α*) and with free TNF-*α* failed to induce significant levels of antibodies compared with IVV alone. The TNF/CHP nanoparticles alone did not induce any measurable antibody response against IVV. Also, we examined antigen specificity further by hemagglutinin- (HA-) specific hemagglutination inhibition (HI) assay because the IVV we used was consisting of different types of influenza virus. The TNF/CHP nanoparticles with IVV induced significant HI activity against all types of HA used (A/H1N1, A/H3N2, and B) (Figure 2(c)). The effects were comparable to those of CTB.

**Figure 3:**
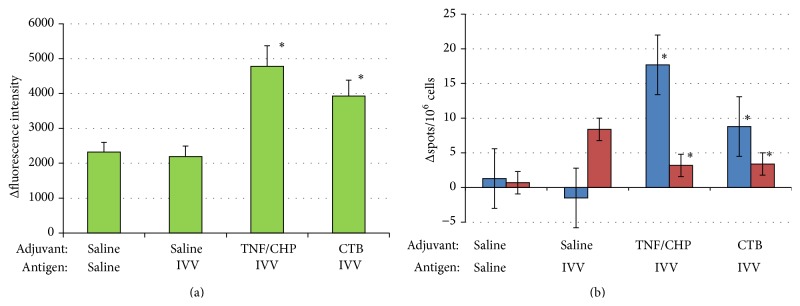
Proliferation and cytokine production by splenocytes from mice nasally administered TNF/CHP nanoparticles. Mice were nasally administered IVV and TNF/CHP nanoparticles or CTB as described. Splenocytes were prepared from the mice and IVV-specific proliferation and IL-4 and IFN-*γ*-producing cells were examined with alamarBlue and ELISpot, respectively. The results represent the difference (Δ) between cases with and without IVV antigen stimulation. (a) Proliferation response, (b) cytokine-producing cells. Blue column, IL-4 production; red column, IFN-*γ* production (mean ± SEM, *n* = 8). ^*∗*^ *P* < 0.05 versus saline/IVV.

Further, the antigen-specific T cell responses of the animals were examined using splenocytes. IVV-specific proliferation in the TNF/CHP nanoparticle group was comparable to that of CTB (Figure 3(a)). Regarding cytokine production as measured by ELISpot, IVV alone increased the IFN-*γ*-producing cells and either adjuvant alone (TNF/CHP nanoparticle or CTB) suppressed the IFN-*γ* production. Instead, IL-4-producing cells were increased by nasal administration of the vaccine and adjuvant combination (Figure 3(b)). The TNF/CHP nanoparticles enhanced host immunity and the effect seemed stronger than that of CTB, a vaccine adjuvant positive control.

### 3.4. Adjuvant Effect of TNF/CHP Nanoparticles in Lethal Challenge of Influenza Virus to Mice

**Figure 4:**
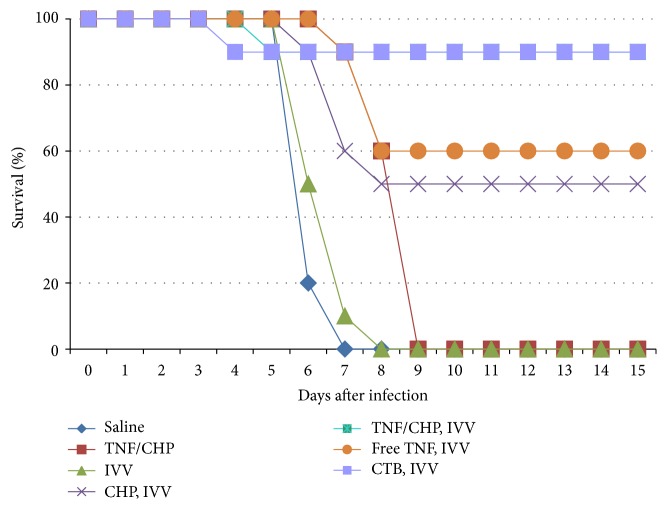
Protective effect of TNF/CHP nanoparticles adjuvant against lethal influenza virus challenge in mice. Mice were nasally administered IVV with or without the adjuvant once a week for 3 times. Seven days after the final immunization, mice were challenged with influenza virus (Puerto Rico/8/34, 10 LD_50_) nasally and the virus-challenged mice were monitored daily. Blue diamond, saline only; red square, TNF/CHP nanoparticles (5 *μ*g/mouse as TNF-*α*) only; green triangle, IVV (0.3 *μ*g/mouse) only; purple cross, blank CHP nanoparticles (240 *μ*g/mouse) with IVV; blue square, TNF/CHP nanoparticles with IVV; orange circle, free TNF (5 *μ*g/mouse) with IVV; and purple dot, CTB (0.8 *μ*g/mouse) with IVV (*n* = 10).

To directly address the stimulatory effect of the TNF/CHP nanoparticles on protective immunity as a vaccine adjuvant, we carried out an experimental lethal influenza virus challenge of immunized mice. The mice were nasally immunized with the IVV (0.3 *μ*g/mouse) combined with or without TNF/CHP nanoparticles (corresponding to 5 *μ*g TNF-*α*/mouse) once a week for 3 times. Mice were challenged with influenza virus A/PR8 strain (160 pfu/mouse, 10 LD_50_) 7 d after the final immunization. The mice that received the IVV only all died by 8 d after the challenge, almost the same as without the IVV immunization. The TNF/CHP nanoparticles without the IVV delayed the onset of death a little, but eventually all the animals died. On the contrary, combined administration of the IVV and the TNF/CHP nanoparticles showed a highly protective effect on the mice and 90% of the animals survived. The effect was comparable to that of CTB as an adjuvant. Free TNF also showed a somewhat protective effect. Interestingly, CHP only (without TNF-*α*) provided a certain level of protection, as we observed up to 50% survival (Figure 4). The surviving animals immunized with the IVV and the TNF/CHP nanoparticles had immunological memory, including IgG_1_ in plasma and IgA in nasal/vaginal wash; this memory was maintained for more than 91 d, and these mice responded to a boosting challenge of the IVV (data not shown). These data indicate that the nanoparticles induced systemic immunity and long-lived memory. Overall, our data demonstrate that TNF/CHP nanoparticles are effective as a vaccine adjuvant for nasally delivered IVV.

### 3.5. Activation of Immune Cells in NALT

**Figure 5:**
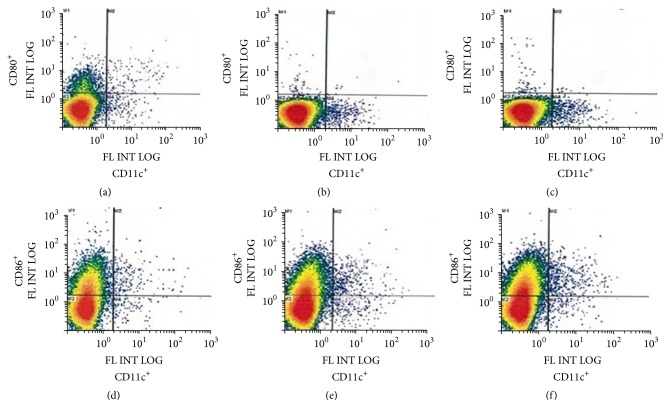
Flow cytometry of NALT cells after immunization with TNF/CHP nanoparticles. Mice were nasally administered with IVV (0.3 *μ*g/mouse) or IVV and TNF/CHP nanoparticles (5 *μ*g/mouse as TNF-*α*) once a week for 3 times. Sixty-eight hours after the last immunization, NALT cells were prepared and subjected to flow cytometric analysis. The expression of CD11c and CD80/CD86 was examined. (a) and (d), saline; (b) and (e), IVV; (c) and (f), IVV with TNF/CHP nanoparticles; (a), (b), and (c), CD80^+^/CD11c^+^; and (d), (e), and (f), CD86^+^/CD11c^+^.

Being focused on the nasal route of vaccination, we examined immune cells in the nasal tissues after the immunization. Sixty-eight h after the nasal immunization 3 times, NALT cells were prepared from mice and expression of surface markers, a marker for DCs (CD11c) and activation markers for B cells (CD80 and CD86), was examined by flow cytometry. The ratio of CD11c^+^/CD80^+^ cell population was 0.20%, 0.30%, and 0.33% for saline, IVV, and IVV with TNF/CHP nanoparticles, respectively. The ratio of CD11c^+^/CD86^+^ population was 0.33%, 0.44%, and 0.50% for saline, IVV, and IVV with TNF/CHP nanoparticles, respectively (Figure 5). Even though the degree was relatively small in extent, IVV vaccination with or without TNF/CHP nanoparticles activated DCs and B cells. But the effect of TNF/CHP nanoparticles was not so prominent.

### 3.6. Antigen Uptake and Activation of the NALT and Nasal Passage Cells

**Figure 6:**
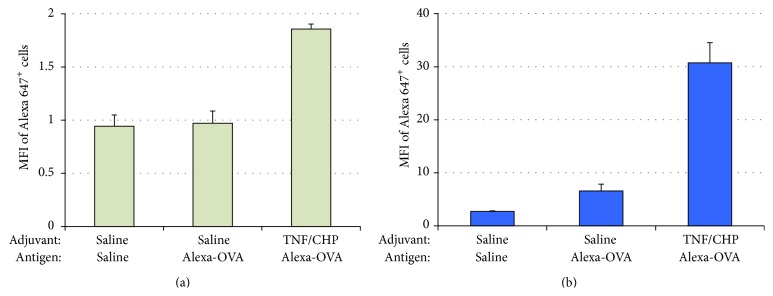
Antigen uptake of NALT and nasal passage DCs after TNF/CHP nanoparticles administration. Mice were immunized for 6 h with 10 *μ*g of Alexa-labeled OVA antigen for nasal administration with or without TNF/CHP nanoparticles as an adjuvant. The NALT and the nasal passage cells were prepared and subjected to FCM analysis with Gatelogic software and antigen uptake was measured by the Alexa 647 florescence intensity for DCs (CD11c^+^ cells) detected in parallel, that is, ratio of Alexa 647^+^/CD11c^+^. (a) NALT DCs, (b) nasal passage DCs. MFI, mean fluorescence intensity (mean ± SD, *n* = 4).

To find out the mechanisms by which the nasally administered TNF/CHP nanoparticles exerted immune-enhancing activity, we next focused on early immune response of nasal mucosal tissues. In these experiments, we used Alexa 647-labeled ovalbumin (OVA) as a model antigen and assessed antigen uptake by DCs in the NALT and nasal passage cells after 6 h of immunization by flow cytometry. The results were analyzed with Gatelogic software and expressed as fluorescence values of Alexa 647^+^/CD11c^+^ cells. Immunization of mice with OVA combined with TNF/CHP nanoparticles activated antigen uptake by both NALT and nasal passage DCs, and the nasal passage DCs responded particularly well. TNF/CHP nanoparticles seemed to stimulate DCs most in the nasal mucosal immune tissue (Figure 6).

### 3.7. Expression of Inflammatory Signals in NALT

**Figure 7:**
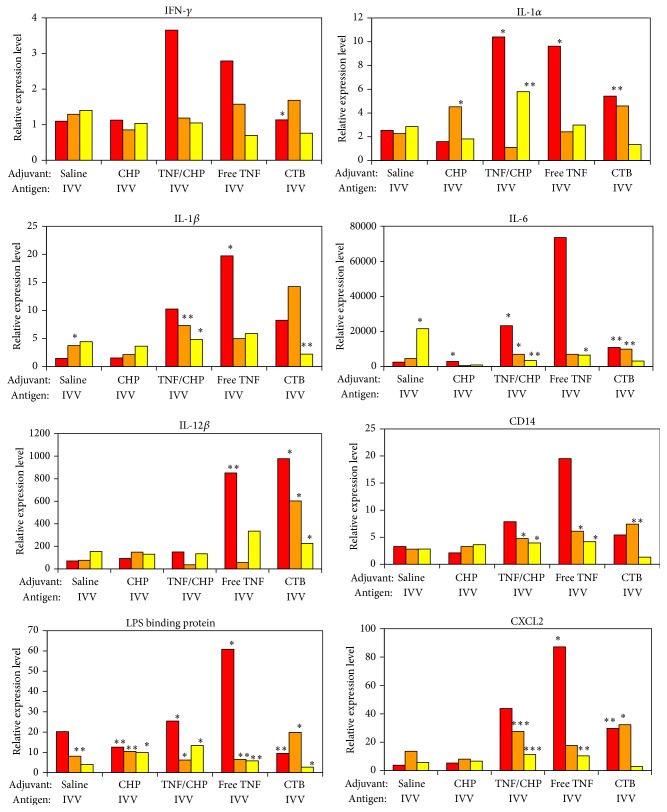
Gene expression in NALT after TNF/CHP nanoparticles administration. mRNA was prepared from NALT cells 2, 6, and 26 h after administration of TNF/CHP nanoparticles and IVV. Gene expression related to innate and adaptive immune responses (Table 1) was analyzed by quantitative PCR, normalized to GAPDH expression. Data are shown as relative level versus control (means of quadruple experiments). Red column, 2 h; orange column, 6 h; and yellow column, 26 h of treatment. ^*∗*^ *P* < 0.05; ^*∗∗*^ *P* < 0.01; ^*∗∗∗*^ *P* < 0.001 versus control.

To understand the nasal tissue activation caused by the TNF/CHP nanoparticles soon after immunization, we conducted gene expression profiling in NALT cells 6 h after nasal immunization with the IVV antigen combined with or without the nanoparticles. By scattering analyses, the gene expression of inflammation-related molecules, for example, triggering receptor expressed on myeloid cells 1, fibronectin-1, CD14, TLR 2, TLR3, IL-1*β*, IL-1 family 9, and IL-6, was found to be significantly upregulated (data not shown). For further analyses of the inflammatory signaling molecules induced by TNF/CHP nanoparticles, we focused on the molecules listed in Table 1 and performed quantitative PCR analyses on 2, 6, and 26 h activated NALT cells. Although CHP itself did not show significant immune-enhancing activity, the inflammatory markers were enhanced when the adjuvants were included (TNF/CHP nanoparticles, free TNF, and CTB). Among the molecules tested, significant increases in the expression of IFN-*γ*, IL-1*α*, IL-1*β*, IL-6, CXCL2, IL-12*β*, CD14, and LPS binding protein were found. The degree of enhancement varied from gene to gene. In particular, the expression of IL-6 and IL-12*β* was intensely enhanced. For these genes, free TNF resulted in strong and early increase in expression. The enhancement tended to be greater at 2 h than 6 or 26 h (Figure 7). Expression profiles with the TNF/CHP nanoparticles were similar to those with free TNF, although the magnitudes were not as great. Although the expression of IL-12*β* was prominent when CTB was used as an adjuvant, the IL-12*β* response was much lower with TNF/CHP nanoparticles.

### 3.8. Preliminary Safety Study of TNF/CHP Nanoparticles

General safety was preliminarily examined. Mice (4 in each sex) nasally administered the TNF/CHP nanoparticles combined with the IVV either once or four times were subjected to an acute and a repeated toxicity study, respectively. The general symptoms, ophthalmic examinations, body weight, body temperature, hematology, blood biochemistry, urinalysis, autopsy findings, and histochemical analyses with hematoxylin-eosin staining were inspected.

#### 3.8.1. General Symptoms, Ophthalmic Examination, Body Weight, and Body Temperature

No general symptoms and behavioral anomaly in either male or female animals were correlated with the TNF/CHP nanoparticles and the IVV treatment during the study period. In ophthalmic examinations, there were no test material-related ocular findings observed. Body weights and body temperatures in the both sexes during the treatment were not statistically different among the treatment groups (data not shown).

#### 3.8.2. Hematology and Blood Biochemistry

Hematology evaluations were also performed during and at the end of study. There were no differences in any of the parameters (white blood cell, red blood cell, hematocrit, lymphocyte, neutrophil, eosinophil, basophil, and monocyte) that were considered to be due to the administration of the TNF/CHP nanoparticles and the IVV. For blood biochemistry, total protein, albumin, urea nitrogen, creatinine, Na^+^, K^+^, Cl^−^, Ca^2+^, inorganic phosphate, aspartate aminotransferase (AST), alanine aminotransferase (ALT), lactate dehydrogenase (LDH), amylase (AMY), *γ*-glutamyl transpeptidase (*γ*-GT), total cholesterol, triglyceride, HDL-cholesterol, total bilirubin, and glucose were examined. All differences found during the study fell within historical control value ranges and were not considered test material-related (data not shown).

#### 3.8.3. Urinalysis

Urobilinogen, bilirubin, ketone body, glucose, protein, pH, specific gravity, nitrite salt, and leucocyte were examined during the study period. There were no TNF/CHP nanoparticles and IVV-related changes observed (data not shown).

#### 3.8.4. Blood Antibody Titer

The anti-IVV IgG_1_ in blood was examined after the administration of TNF/CHP nanoparticles and IVV. No detectable antibody was induced by the single administration. After the second administration, however, the antibody was induced by only the TNF/CHP nanoparticles combined with the IVV and the titer was maintained even after 2 weeks of cessation period. The effectiveness of the TNF/CHP nanoparticles as vaccine adjuvant was confirmed. In this experiment, females tended to respond to the immunization higher (Table 3).

#### 3.8.5. Pathology and Major Organ Weights

Gross pathology of all the animals was examined at the end of the study. There were few gross pathology finding and none of them were considered to be the TNF/CHP nanoparticles and IVV-related. The major organs (brain, heart, lung, kidney, liver, ovary, testis, spleen, adrenal, and thymus) were measured at the end of the study in all of the animals. No organ weight change was noted as test material-related (data not shown).

#### 3.8.6. Histopathology

Histopathology of tissues from animals in each group was examined and there were few histopathological findings, and none of them were considered to be TNF/CHP nanoparticles and IVV-related. As the nasal administration, possible harmful effects on central nervous system were concerned. However, no abnormalities in the brain, especially olfactory bulb, were noted in any animals after histological examination.

**Figure 8:**
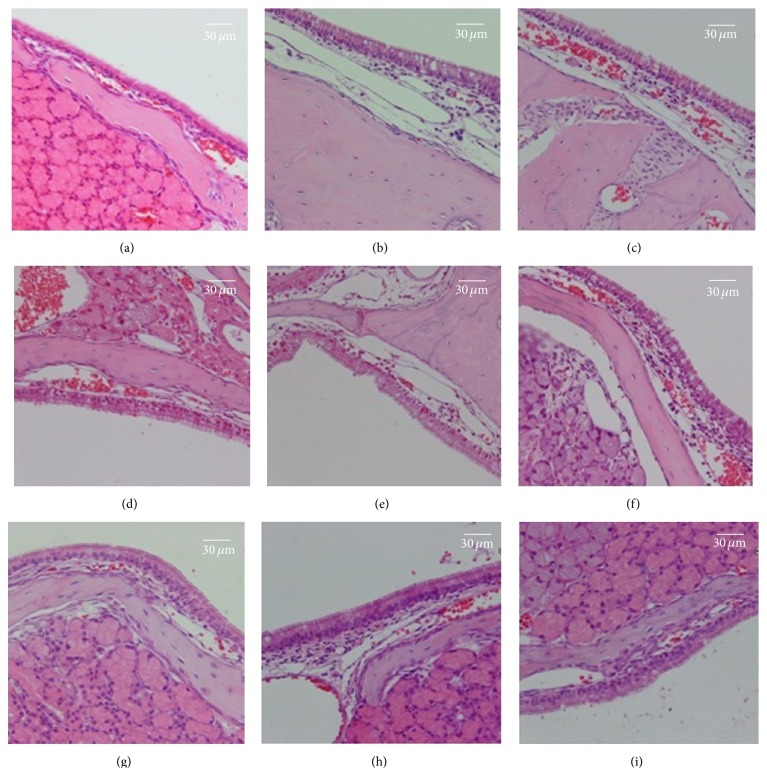
Histopathology of nasal mucosal tissue by TNF/CHP nanoparticles administration. Nasal tissues of mice administered TNF/CHP nanoparticles were isolated, stained with hematoxylin-eosin, and inspected. Representative histopathological findings are shown. (a), (b), and (c), 2 days after administration once; (d), (e), and (f), 2 days after repeated administration of once a week for 4 times; (g), (h), and (i), repeated administration of once a week for 4 times followed by 2 weeks of cessation period; (a), (d), and (g), saline; (b), (e), and (h), saline and TNF/CHP nanoparticles (5 *μ*g/mouse as TNF-*α*); (c), (f), and (i), IVV (0.3 *μ*g/mouse) and TNF/CHP nanoparticles (5 *μ*g/mouse as TNF-*α*); and bar, 30 *μ*m.

On the other hand, there were some influences observed in nasal mucosal tissue. First, single administration of any specimen showed no effects. In the case of repeated administration, IVV did not affect the mucosal tissues greatly except slight infusion probably caused by repeated stimulation. On the other hand, TNF/CHP nanoparticles combined with IVV induced slight-to-moderate infusion and infiltration of inflammatory cells (lymphocytes, neutrophils, eosinophils, and mast cells). The findings, however, diminished in trace proportions after 2 weeks of cessation period. No excessive inflammatory symptoms, such as formation of edema or fibrosis, were noted (Figure 8).

In summary, no significant change due to the administration of the TNF/CHP nanoparticles and IVV was observed in the acute toxicity study. In the repeated toxicity study, slight-to-moderate infusion and inflammatory cell infiltration were observed at the nasal mucosa, the administration site. This response, however, seemed reversible. Overall, no immunotoxicity was detected. Although further evaluation is required, our results demonstrated that the toxicity of TNF/CHP nanoparticles is relatively low.

## 4. Discussion

In this study, we attempted to test a new adjuvant preparation with a potent immunoregulatory cytokine and a DDS material. Human TNF-*α* was successfully incorporated into CHP complexes to form stable nanoparticles. The preparation process was efficient and the resulting nanoparticles were relatively uniform (Figure 1) and robust. The nanoparticles had excellent storage stability for more than 3 weeks at room temperature (Table 2). Many medical formulations, especially biological ones, require storage at low temperatures or freezing. On the contrary, the TNF/CHP nanoparticles could be stored in solution and without refrigeration. Our formulation offers improved convenience of handling and transportation.

Me-*β*-CD is known to interact with cholesteryl groups and disrupt CHP complexes to release the substance inside the particles [21]. In the case of the TNF/CHP nanoparticles, the amount of Me-*β*-CD required to disrupt the particle structure was approximately 100 mg/mL, much higher than 0.3 mg/mL reported in the case of IL-12/CHP nanoparticles [21], suggesting that the affinity between TNF-*α* and CHP was much stronger than that of IL-12 and CHP. It seems that TNF-*α* trimers fit well in the inner space of the CHP complex. The molecular interactions of TNF-*α* and CHP are a topic of further study for our group.

We examined the immune-enhancing activity of the TNF/CHP nanoparticles administered nasally. When administered the IVV, TNF/CHP nanoparticles induced high levels of IgA in the nasal wash, as well as IgG_1_ in blood plasma (Figures 2(a) and 2(b)). Furthermore, the antigen-specific antibody response was induced against influenza type B as well as type A/H1N1 and type A/H3N2, which consisted in the IVV used (Figure 2(c)). It is suggested that nasal vaccination covers broad range of antigenicity as reported [3, 4]. The effects were comparable to those of CTB, which is recognized to be the most powerful vaccine adjuvant in experimental settings [31]. These data indicate that TNF/CHP nanoparticles administered nasally can induce not only mucosal but also systemic immunity significantly and efficiently. The stimulatory effects were seen with other antigens, such as Hepatitis virus type A vaccine and diphtheria toxoid (data not shown). These data suggest that TNF/CHP nanoparticles have the potential as a vaccine adjuvant with a broad range of applications.

TNF/CHP nanoparticles elicited immune activation comparable to that of CTB (Figure 3(a)). ELISpot analysis indicated that the nanoparticles decreased IFN-*γ*-producing cells and increased IL-4-producing cells (Figure 3(b)). These data suggest that the nasally administered adjuvant shifted the Th1/Th2 balance to a Th2-dominant state and confirmed previous results with a mutant TNF-*α* [15]. The effects of the TNF/CHP nanoparticles seemed stronger than those of CTB.

To directly address the use of TNF/CHP nanoparticles as a vaccine adjuvant on protective immunity, we employed a lethal influenza challenge mouse model. The mice were immunized with influenza virus A/Brisbane/59/2007 (H1N1), A/Uruguay/716/2007 (H3N2), and B/Brisbane/60/2008, followed by challenge with an antigenically distinct influenza virus A/Puerto Rico/8/34 (H1N1). The nasally administered TNF/CHP nanoparticles induced protective immunity in spite of the distinct antigenicities [32], suggesting that they have a potential for inducing broad cross-protection (Figure 4). Muraoka et al. proposed that CHP-based nanoparticles preferentially deliver the antigen to antigen-presenting cells in lymph nodes to potentiate effective immune responses [33]. This might be the reason why the TNF/CHP nanoparticles induced excellent protective immunity. They, however, reported that CHP itself did not show an adjuvant effect in the context of a tumor vaccine [25]. Interestingly, CHP only (without TNF-*α*) showed a certain level of efficacy in our study. The reason for the discrepancy between their results and ours is not clear. It is unlikely that TNF-*α* was replaced* in vivo* by IVV antigens to form IVV/CHP nanoparticles, which would then preferentially deliver vaccine antigens to the lymph node given the short time frame. Further, Oyewumi et al. reported that particle size was critical for adjuvant activity [34]. Because the DLS analyses showed no difference in particle size between the TNF/CHP nanoparticles and empty CHP particles (Figure 1), the size difference between them does not explain the discrepancy, either. Protection against external pathogens such as influenza virus and internal antigens such as tumor antigen might rely on different mechanisms.

TNF/CHP nanoparticles enhanced an IgA response not only at the site of application (i.e., in the nasal wash) but also at distant mucosal sites, including vaginal and salivary glands (data not shown). IgA antibody elicited at the mucosa is of vital importance as the natural route of infection for influenza is via the respiratory mucosa. Hence, local mucosal protection against pharyngeal carriage is likely to be decisive for preventing disease [35]. Conventional parenteral vaccines are not able to stimulate mucosal immune responses, thus restricting their efficacy in infections of mucosal surfaces such as the respiratory tract [3]. Our nasal vaccine/adjuvant formulation consisting of the IVV and TNF/CHP nanoparticles effectively induced both systemic and mucosal protective immunity. Antibodies became detectable after the second or the third vaccination and reached plateau levels thereafter in mice vaccinated with TNF/CHP nanoparticles (data not shown). Also, the nanoparticles maintained immune responses for a long period of time, at least for 91 d (data not shown). These data indicate that nanoparticles induced long-lived immune memory, a critical feature for successful vaccine adjuvants.

The mucosal surfaces are known to have abundant B cells, T cells, and plasma (or DC) cells. After the repeated immunization of animals, activation of DC and B cells seemed enhanced in NALT tissue by TNF/CHP nanoparticles, despite being in small extents (Figure 5). Uptake of antigen by the mucosal tissues is essential for the induction of immune responses [36]. Therefore, we examined antigen uptake by NALT-resident and nasal passage DCs, the inductive sites of common mucosal immune system (CMIS) [37]. TNF/CHP nanoparticles seemed to stimulate NALT and the nasal passage DCs, in particular the latter more intensely (Figure 6). Also, TNF/CHP nanoparticles enhanced expression of DC and B cell activation markers (CD40, CD80, and CD86) in bone marrow-derived immature DC preparation (data not shown). Considering a relatively large volume of the nasal administration (30 *μ*L/mouse) in this study, a possibility that TNF/CHP nanoparticles affect immune cells in the lower respiratory tract as well as nasal mucosa should be considered. We plan to explore the possibility in future studies.

Vaccine adjuvants trigger the innate immune system to enhance humoral and cellular responses to the coadministered vaccine antigens. To understand the mechanisms by which TNF/CHP nanoparticles activate innate immunity, we conducted gene expression profiling in NALT cells. The expression of genes related to inflammation and immunity was found to be upregulated (data not shown). Based on these results, we focused on the inflammatory signaling molecules and performed quantitative PCR analyses. Enhanced expression of IL-1*α*, IL-1*β*, IL-6, CXCL2, IL-12*β*, CD14, and LPS binding protein was found in TNF/CHP nanoparticle-administered mice. Free, exogenous TNF elicited strong and early increases in the expression of inflammatory signaling molecules. In comparison, the pattern was similar but the extent was less with TNF/CHP nanoparticles (Figure 7). The nanoparticles might effect a slow release of TNF-*α*, prolonging the immune-stimulatory effect. Taken together, TNF/CHP nanoparticles seemed to protract activation of innate immunity. The nanoparticles might prolong the stimulatory effect of TNF-*α* moderately and thereby provide the immune-enhancing adjuvant effect. Further, although expression of IL-12*β* was elicited in response to the CTB adjuvant, IL-12*β* was weakly elicited in the case of TNF/CHP nanoparticles. CHP seemed to minimize the unfavorable effects of TNF-*α* while promoting its beneficial activities. One important issue related to the development of nasal vaccines is safety concerns about the potential dissemination of vaccine antigens to the central nervous system (CNS). Past reports suggested that nasal administration of CTB reached the CNS and accumulated in olfactory tissues. It caused Bell's Palsy in clinical studies, probably due to IL-12 production, and the use of CTB in humans was prohibited [38, 39]. In that context, lower expression level of IL-12*β* might be a beneficial safety feature of TNF/CHP nanoparticles.

Although CHP itself did not show immune-enhancing activity such as increasing IgG_1_ and IgA or the expression of inflammation-related genes, it showed a certain level of protection in a lethal influenza virus challenge (Figure 4). We cannot easily account for this observation. There are likely other pathways and mechanisms involved and waiting to be clarified.

Very recently, Onishi et al. reported that hydroxypropyl-*β*-cyclodextrin (HP-*β*-CD), another type of saccharide-based material that can form nanoparticles, exhibited adjuvant activity and elicited a strong protective effect against influenza virus in mice and cynomolgus* macaques* [40]. They suggested the involvement of Tfh cells via MyD88- and TBK-dependent pathways. Their findings may shed some light on additional mechanisms at play with nanoparticles as vaccine adjuvants. However, they mentioned the cytotoxicity of HP-*β*-CD at more than 0.5%* in vitro*, probably because of *β*-CD's ability to extract cholesterol out of cell membranes [41]. TNF/CHP nanoparticles might represent a preferable alternative.

Preliminary safety studies revealed no severe toxic findings resulting from the exposure to TNF/CHP nanoparticles. In the repeated toxicity study, slight-to-moderate infusion and infiltration of inflammatory cells were observed at nasal mucosal tissue, the administration site. This response would likely be reversible over time. And enhancing effect of TNF/CHP nanoparticles in the case of repeated administration on induction of antigen-specific antibody in blood was confirmed again (Table 3). Even through the careful histopathological examination, no abnormal finding in brain including olfactory bulb was noted, suggesting no obvious damage of the CNS. No obvious immunotoxicity was detected and the results suggested that TNF/CHP nanoparticles are relatively safe as a nasal vaccine adjuvant.

## 5. Conclusions

The results of this study demonstrate that TNF/CHP nanoparticles are effective as a vaccine adjuvant when administered via the nasal mucosal route. Moreover, the ability of TNF/CHP nanoparticles to stimulate comparatively balanced systemic as well as mucosal immune responses makes them a potentially promising vaccine adjuvant for inducing immunity against infectious pathogens. In the short term, TNF/CHP nanoparticles may provide a useful way for developing new nasal influenza vaccines. Further, we propose that combining TNF/CHP nanoparticles with next-generation vaccine platforms that do not rely on the cold chain will offer valuable alternatives for vaccination in a variety of settings.

## Figures and Tables

**Table 1:** Primer sets for quantitative PCR.

Name	Genbank accession number	Forward	Reverse	Length/Tm (bp/°C)
IFN-*γ*	NM_008337	tcaagtggcatagatgtggaagaa	tggctctgcaggattttcatg	92/60
IL-4	NM_009841	acaggagaagggacgccat	gaagccctacagacgagctca	95/60
IL-1*α*	NM_010554	atgtatgcctactcgtcggg	tgagttttggtgtttctggc	139/60
IL-1*β*	NM_008361	caaccaacaagtgatattctccatg	gatccacactctccagctgca	152/60
IL-2	NM_008366	cctgagcaggatggagaattaca	tccagaacatgccgcagag	141/60
IL-5	NM_010558	agcacagtggtgaaagagacctt	tccaatgcatagctggtgattt	117/60
IL-6	NM_031168	agttgccttcttgggactga	ttgccattgcacaactcttt	186/60
IL-10	NM_010548	ggttgccaagccttatcgga	acctgctccactgccttgct	191/62
IL-12*β*	NM_008352	agctcgcagcaaagcaaggt	tggagacaccagcaaaacga	181/62
IL-17A	NM_010552	gctccagaaggccctcaga	agctttccctccgcattga	142/60
IL-18	NM_008360	ccaaatcacttcctcttggc	ggccaaagttgtctgattcc	144/60
IFN-*β*1	NM_010510	ccgagcagagatcttcaggaa	cctgcaaccaccactcattct	106/60
fibronectin 1	NM_010233	gggagaagtttgtgcatggt	ctgggggtctccgtgataat	136/60
CD14	NM_009841	catttgcatcctcctggtttctga	gagtgagttttccccttccgtgtg	182/55
proteoglycan 2	NM_008920	acttgacaagacccaggagg	ctcatccatcaatgggcttt	135/60
LPS binding protein	NM_008489	tcgccatctctgactcttcc	ggaggtccactgaaatggtg	120/60
Lactotransferrin	NM_008522	aatccaatctctgtgccctg	atgcaacatttcctgccttc	132/60
TLR3	NM_126166	tgcagtctttccagagggat	acaaaagtcccccaaaggag	133/60
CXCL2	NM_009140	gagcttgagtgtgacgcccccagg	gttagccttgcctttgttcagtatc	148/58
TNF-*α*	NM_013693	cgtcgtagcaaaccaccaag	gtgggtgaggagcacgtagt	187/62
GAPDH	NM_002046	accatcttccaggagcgag	agtgatggcatggactgtgg	324/60

**Table 2:** Stability of TNF/CHP nanoparticles *in vitro*.

	M-*β*-CD	Days			
		0	7	14	21
TNF-*α* (*µ*g/mL)	−	10	2	1	1
	+	76	65	78	72

TNF/CHP nanoparticles were incubated in D-PBS at room temperature. An aliquot was examined for the amount of active TNF-*α* by ELISA. Samples were treated with 100 mg/mL M-*β*-CD at 37°C for 2 h to release TNF-*α* from the particles. The values are means of triplicate experiments.

**Table 3:** Antibody titer in blood plasma.

	Anti-IVV antibody titer in plasma (U/mL)					
	Male			Female		
Adjuvant	Saline	TNF/CHP	TNF/CHP	Saline	TNF/CHP	TNF/CHP
Vaccine	Saline	Saline	IVV	Saline	Saline	IVV
Week						
1	n.d.	n.d.	n.d.	n.d.	n.d.	n.d.
2	n.d.	n.d.	140.0 ± 93.0	n.d.	n.d.	292.0 ± 146.0
4	n.d.	n.d.	1807.0 ± 795.0	n.d.	n.d.	2334.0 ± 609.0
4 + 2	n.d.	n.d.	7602.0 ± 2265.0	n.d.	n.d.	10695.0 ± 4045.0

The vaccine (IVV 0.3 *µ*g/mouse) and adjuvant preparation (TNF/CHP nanoparticles 5 *µ*g/mouse as TNF-*α*) were nasally administered to mice once a week up to 4 weeks. After that, 2 weeks of cessation period was set (4 + 2 weeks). Blood plasma was prepared and the anti-IVV IgG_1_ antibody was titrated (*n* = 4). n.d., not detected.
